# Gamma Radiation Synthesis of Ag/P25 Nanocomposites for Efficient Photocatalytic Degradation of Organic Contaminant

**DOI:** 10.3390/nano13101666

**Published:** 2023-05-18

**Authors:** Zihua Zeng, Shuangxiao Li, Xueyan Que, Jing Peng, Jiuqiang Li, Maolin Zhai

**Affiliations:** Beijing National Laboratory for Molecular Sciences, Radiochemistry and Radiation Chemistry Key Laboratory of Fundamental Science, The Key Laboratory of Polymer Chemistry and Physics of the Ministry of Education, College of Chemistry and Molecular Engineering, Peking University, Beijing 100871, China

**Keywords:** Ag/P25 nanocomposites, gamma-ray radiation method, photocatalytic performance

## Abstract

Titanium dioxide (TiO_2_) has garnered significant attention among various photocatalysts, whereas its photocatalytic activity is limited by its wide bandgap and inefficient charge separation, making the exploration of new strategies to improve its photocatalytic performance increasingly important. Here, we report the synthesis of Ag/P25 nanocomposites through a one-step gamma-ray radiation method using AgNO_3_ and commercial TiO_2_ (Degussa P25). The resulting products were characterized by powder X-ray diffraction, UV-Vis diffused reflectance spectroscopy, transmission electron microscopy, and X-ray photoelectron spectroscopy. The effect of free radical scavengers, feed ratios of Ag/P25, and dose rates on the photocatalytic activity of the Ag/P25 nanocomposites were systematically investigated using rhodamine B under Xenon light irradiation. The results showed that the Ag/P25 photocatalyst synthesized with a feed ratio of 2.5 wt% and isopropyl alcohol as the free radical scavenger at a dose rate of 130 Gy/min exhibited outstanding photocatalytic activity, with a reaction rate constant of 0.0674 min^−1^, much higher than that of P25. Additionally, we found that the particle size of Ag could be effectively controlled by changing the dose rate, and the Ag/P25 nanocomposites doped with smaller size of Ag nanoparticles performed higher photocatalytic activities. The synthesis strategy presented in this study offers new insight into the future development of highly efficient photocatalysts using radiation techniques.

## 1. Introduction

Environmental protection has become an urgent and crucial concern nowadays in the face of increased organic and inorganic pollutants in the natural environment due to rapid industrialization [1,2]. The consequences of this pollution pose a significant threat to human health, social stability, and sustainability [3,4]. Consequently, there has been a surge of research in both academic and industrial settings toward reducing environmental pollution from organic and inorganic pollutants. The challenge lies in finding simple, eco-friendly, and effective techniques to remove contaminants [5].

Advanced oxidation processes (AOPs) are a series of chemical treatment for water or wastewater aimed at removing organic contaminants by oxidation. AOPs rely on the in situ generation of highly reactive oxidants, in particular, hydroxyl radical (OH·), which can oxidize virtually any compound in water [6]. The applications of AOPs in the treatment of a wide range of organic pollutants such as industrial dyes, pesticides, and fertilizers, have shown high reaction rate constants and excellent removal efficiency [7,8]. Photocatalysis, as a process technology which has been investigated for use as a kind of AOPs, has emerged as a promising and eco-friendly method, owing to its energy-saving, environmentally friendly, and highly efficient properties [9,10,11]. During the photocatalytic process, reactive oxygen species (ROS) are generated from electron capture by oxygen and water oxidation, such as superoxide (O_2_**^−^**) and OH·, which will engage in subsequent oxidation–reduction reactions as highly reactive oxidants [12]. Unlike activated carbon adsorption or membrane filtration methods, photocatalysis can achieve complete degradation of organic and inorganic pollutants through a series of oxidation–reduction reactions [13,14].

Photocatalysts play a crucial role in the photocatalytic process since they greatly affect the absorbance spectrum and the electron–hole separation efficiency, which ultimately determines the photocatalytic efficiency [15]. Semiconductors have been extensively studied as photocatalysts because of their unique energy band structures and their ability to contain inequal numbers of electrons and holes [16,17]. The P-type semiconductors contain an excess of holes, while the N-type semiconductors are abundant of electrons. As a N-type semiconductor, titanium dioxide (TiO_2_) has garnered significant attention due to its low cost, easy commercial availability, nontoxicity, high thermal and photochemical stability, and unique optical properties [18,19]. TiO_2_ exists in various nanoscale forms, including nanospheres [20], nanowires [21,22], nanotubes [23], nanofibers [24], and has found wide-ranging applications in pollutant degradation [25,26], hydrogen production [27,28], and carbon dioxide reduction [29], etc. However, the large band gap width of TiO_2_ (3.0–3.2 eV) restricts its light absorption to the UV region and limits its use in visible light irradiation [13,30]. Additionally, TiO_2_ exhibits fast recombination of photogenerated charges, which decreases the generation of ROS, lowers the photocatalytic quantum yield for OH· radical production, and hence hampers its practical application in photocatalysis [31].

To overcome these challenges, researchers have employed a range of specific surface modifications, including doping with non-metallic or metallic elements [27,32,33,34,35] and compounding with other semiconductors [14,36,37]. Moreover, the Z-scheme, a conceptual model in photosynthesis, has inspired researchers to design heterostructures and hybrid systems to preserve photogenerated electrons and holes with higher redox potentials and improve photocatalytic performance [38]. Among these modification methods, doping noble metal nanoparticles onto the surface of TiO_2_ has proven particularly effective because the doped metal nanoparticles usually have high Schottky barriers, functioning as electron traps to enhance electron–hole separation and inhibit recombination of electrons and holes at the same time [39,40]. Additionally, the localized surface plasmon resonance (LSPR) effect triggered by the doped metal nanoparticles reduces the band gap width of metal-modified TiO_2_, extending its absorption range into the visible light spectrum [41,42]. Silver (Ag) nanoparticles offer advantages over other noble metals, including ease of availability, excellent conductivity [43], and anti-bacterial properties [44]. Recent studies have focused on controlling the shapes and sizes of metal nanoparticles, including Ag. Under certain experimental conditions, the size of Ag nanoparticles can be controlled [45,46], influencing the LSPR effect, similar to other metals such as platinum [47], aurum [48], and ruthenium [49].

Various techniques have been developed to prepare Ag/TiO_2_ nanocomposites, including photoreduction [50], sol-gel [51], solvothermal and hydrothermal treatment [52,53,54,55,56], as well as plasmonic fabrication [57,58,59,60]. In the field of water purification, extensive studies have demonstrated that Ag/TiO_2_ nanocomposites can be applied to photodegrading organic dyes such as rhodamine B (RhB). Liang et al. synthesized Ag/TiO_2_ nanocomposites under UV light irradiation and proposed different mechanisms of selective oxidation of RhB under UV or visible light [61]. Zhou et al. fabricated heterostructured Ag/g-C_3_N_4_/TiO_2_ ternary photocatalyst through thermal oxidation etching process to form g-C_3_N_4_/TiO_2_ and photoreduction process to load Ag nanoparticles on it. The as-synthesized photocatalyst showed a reaction rate constant of 0.0179 min^−1^ when degrading RhB under visible light, and high recycling stability [62]. As a matter of fact, the synthesis of the aforementioned Ag/TiO_2_ nanocomposites necessitates intricate experimental conditions or relatively elaborate procedures. For instance, both solvothermal and hydrothermal treatments demand the sample to be heated above 180 °C for 5–10 h, while plasmonic fabrication requires intricate pretreatment to establish a suitable atmosphere for plasma treatment.

Radiation synthesis, however, presents a promising technology for synthesizing Ag/TiO_2_ nanocomposites in aqueous solutions, since it is a clean, simple one-step, and highly effective method [63]. In the gamma-irradiation process, both oxidative and reductive species are generated such as electrons, H· and OH· radicals, where OH· radicals perform strong oxidative property, while the electrons and H· radicals possess the reductive potential to reduce Ag(I) [64,65]. This process can be carried out at room temperature without side reaction products, and the radiation chemical yield of those reductive species can usually achieve a high value, indicating its high efficiency. A free radical scavenger, which can prevent oxidative radicals from oxidizing the reduced Ag nanoparticles and reduce excess of reductive radicals, is essential to this method. Most common used free radical scavengers are alcohols (i.e., isopropyl alcohol [49], tert-butyl alcohol [66], and ethanol glycol [67]) and formic acid [68], and imidazolium-based ionic liquids also have diverse applications due to their high radiation stability [69]. Additionally, the irradiation dose rate plays an important role in radiation synthesis, since different dose rates affect the size of nanoparticles during their formation [63], and Ag particle size is controllable by changing different dose rate. To date, there have been few studies on the radiation synthesis of Ag/TiO_2_ nanocomposites, particularly with regard to how dose rate influences the photocatalytic activities.

Here, we report the synthesis of Ag/P25 nanocomposites through a one-step gamma-ray radiation method using AgNO_3_ and commercial TiO_2_ (Degussa P25). Rhodamine B was selected as the model organic contaminant to investigate the photocatalytic performances of the resulting Ag/P25 nanocomposites under Xenon light irradiation. The effect of Ag/P25 feed ratio, dose rate, and different free radical scavengers on photocatalytic activity and the related mechanism were both discussed.

## 2. Materials and Methods

### 2.1. Materials

TiO_2_ (P25, Degussa, Germany), silver nitrate (AgNO_3_, AR, Xilong Scientific Co., Ltd., Shantou, China), isopropyl alcohol (IPA, AR, Beijing TongGuang Fine Chemicals Company, Hebei, China), ethylene glycol (EG, AR, Concord, Tianjin, China), 1-ethyl-3-methylimidazolium acetate (EMImAc, 99%, Energy Chemical, Shanghai, China), rhodamine B (RhB, AR, Macklin, China). All reagents were used as received without further purification.

### 2.2. Synthesis of Ag/P25 Nanocomposites

Ag/P25 nanocomposites were synthesized through a one-step gamma-ray radiation reduction method. In detail, 200 mg P25 powders were added to a mixed solution of IPA and pure water (1:9, *v*/*v*, 40 mL in total). The resulting suspensions were sonicated for 30 min to make P25 powders evenly dispersed. Different volumes of AgNO_3_ solution (0.020 mol/L) were injected into the as-prepared suspensions to form a series of hybrid solutions with different feed ratios of Ag to P25 (0.5 wt%, 1.0 wt%, 2.5 wt%, and 5.0 wt%, respectively). The samples were then bubbled with nitrogen for 10 min to remove oxygen, sealed, and irradiated by gamma-ray from a ^60^Co radiation source (Department of Applied Chemistry of Peking University). The absorbed dose was fixed at 28 kGy with different dose rates (7.2 Gy/min, 31 Gy/min, and 130 Gy/min). The products were subsequently centrifuged, washed with pure water several times, and finally lyophilized for three days. Two additional samples were synthesized under the conditions of 2.5 wt% Ag/P25 mass ratio and a dose rate of 31 Gy/min, with the pre-solutions mixed with 1.0327 g ionic liquid EMImAc (1 mL) and 39 mL pure water, or EG and pure water (1:2, *v*/*v*, 40 mL in total). The postprocessing of products remained unchanged. The abbreviations of the prepared samples and details of their synthesis are listed in Table 1.

### 2.3. Characterization

The phase compositions were analyzed by powder X-ray diffraction (XRD, PANalytical, Netherland, X-Pert3 Powder) with Cu Kα radiation (λ = 1.5418 Å). The X-ray photoelectron spectroscopy (XPS) spectra were recorded on the AXIS Supra X-ray photoelectron spectrometer (Kratos Analytical, Manchester, UK) with an exciting source of Al Kα, using adventitious carbon (C 1s = 284.80 eV) as the calibration reference. The UV-vis diffuse reflectance spectra were measured by a UV-3600 Plus ultraviolet-visible-NIR spectrometer (Shimadzu, Nagoya, Japan) with the wavelength ranging from 200 to 800 nm. The micro morphologies of the catalysts were studied by field emission high-resolution transmission electron microscopy (HRTEM, JEOL, Tokyo, Japan, JEM-2100) at an acceleration voltage of 200 kV. The actual mass ratios of Ag in catalysts were characterized by inductively coupled plasma-optical emission spectrometry (ICP-OES, Leeman, Northern Outagamie County, WI, USA, Prodigy 7).

### 2.4. Measurements of Photocatalytic Activities

The photocatalytic activity of the Ag/P25 nanocomposites was estimated by the photodegradation of RhB (as the target pollutant) under Xenon light irradiation. To this end, a 100 mL jacketed beaker was used as the photoreactor. In the typical experiment, 30 mg catalyst was dispersed in the beaker containing a 90 mL aqueous solution of 30 mg/L RhB (pH = 5.5~6.5). The suspension was sonicated for 5 min and stirred for 55 min in the dark to reach an absorption–desorption equilibrium of RhB. Subsequently, the mixed suspension was exposed to Xenon light irradiation for 90 min while the distance between the lamp and the top surface of the suspension remained unchanged. At predetermined time intervals, a 2.5 mL aliquot was drawn out from the suspension and filtered by a 0.22 μm Nylon filter. Afterward, the transparent solution was transferred to a quartz cuvette and analyzed by UV-Vis absorption spectroscopy in a wavelength range from 450 nm to 600 nm. The concentration of RhB was determined by the absorbance at the wavelength of 554 nm, in accordance with the Lambert–Beer’s Law. To investigate the photocatalytic recycling stability of the catalyst, a 12 mg sample was added to a 36 mL aqueous solution of 30 mg/L RhB. The suspension was irradiated with the Xenon light for 60 min. After filtration and washing, the catalyst was retrieved and added to another RhB solution with the same conditions, and this process was repeated three times. In this photocatalytic process, only the initial and final concentrations were measured to calculate the removal percentage of RhB in the solution.

The degradation rates of RhB with Ag/P25 nanocomposites are calculated by the following formula:

Degradation (%) = (*c*_0_ − *c_t_*)/*c*_0_ × 100 = (*A*_0_ − *A_t_*)/*A*_0_ × 100, (1)

where *c*_0_ is the initial concentration of RhB solution and *c_t_* is the corresponding concentration of RhB solution at time *t*, *A*_0_ is the initial absorbance of RhB solution and *A_t_* is the corresponding absorbance of RhB solution at time *t*. The reaction rate constant k is calculated based on a pseudo-first order kinetics [61] from the following equation:

−ln(*A_t_*/*A*_0_) = −ln(*c_t_*/*c*_0_) = k*t*. (2)

## 3. Results and Discussion

### 3.1. Synthesis and Characterizations of Ag/P25 Nanocomposites

**Figure 1:**
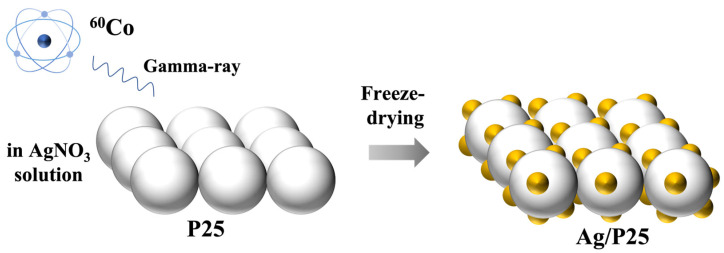
Schematic illustration of the one-step synthesis of Ag/P25 nanocomposites.

**Figure 2:**
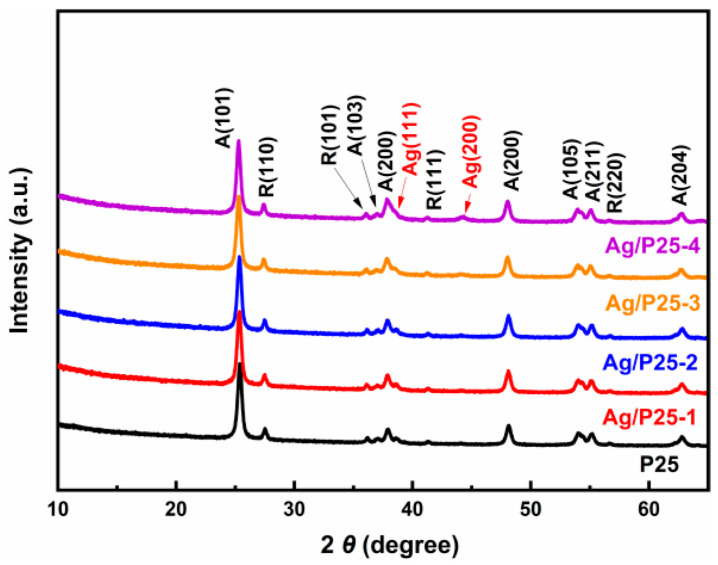
PXRD patterns of Ag/P25 nanocomposites of different Ag/P25 feed ratios. A: anatase TiO_2_ (21-1272); R: rutile TiO_2_ (21-1276); Ag: cubic silver (04-0783).

Ag/P25 nanocomposites were synthesized through a simple one-step gamma-ray induced reduction method, as illustrated in Figure 1. After being irradiated by gamma-ray from ^60^Co source, Ag(I) was reduced to Ag nanoparticles (NPs) and doped on the surface of P25. The conversion to Ag NPs is confirmed by PXRD results. As shown in Figure 2, the peak at 44.3° can be attributed to (200) crystal plane of the cubic Ag phase (04-0783) of Ag/P25-4. The weak intensity of the Ag diffraction peak in Ag/P25-1 was due to the small amount of AgNO_3_ precursor added, and the detection limitation of the PXRD technique. The ICP-OES results presented in Table 2 provide the actual mass ratios of Ag/P25 for the samples, which demonstrate an increase corresponding to the feed ratios of Ag/P25. Notably, when the sizes of Ag NPs are similar (1~3 nm), their actual amounts also show a proportionality to their respective feed ratios [70]. Furthermore, under the conditions studied, the doping efficiency of Ag on P25 consistently exceeds 70%, thereby indicating the efficiency of the method.

**Figure 3:**
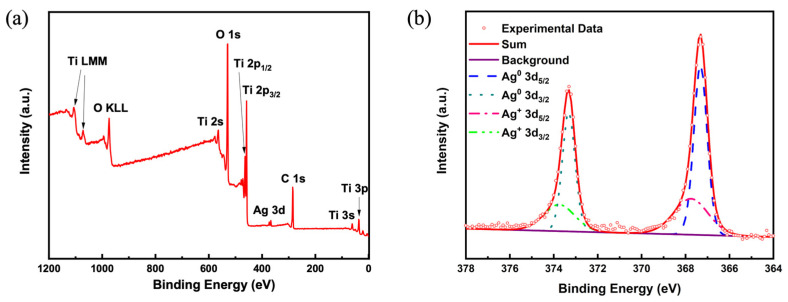
(**a**) XPS spectra survey scan of Ag/P25-6; (**b**) Ag 3d XPS spectrum of Ag/P25-6.

To further confirm the elementary components of the catalysts and the valence state of Ag inside, XPS spectra of Ag/P25-6 are obtained and shown in Figure 3. The survey scans of XPS spectra (Figure 3a) indicate that Ag/P25 nanocomposites mainly consist of Ag, Ti, O, C, and no other elements, which is consistent with the results of PXRD. The XPS spectrum of C 1s (not shown) is used for the purpose of the drift correction of other elements. The XPS spectrum of Ag 3d in Figure 3b shows fitted peaks at 373.3 eV and 367.3 eV, which are ascribed to Ag^0^ 3d_3/2_ and Ag^0^ 3d_5/2_, respectively. These two peaks with a 6.0 eV splitting confirm the existence of metallic Ag particles on P25 through the reduction of Ag^+^ during the irradiation process. The fitted peaks corresponding to Ag^+^ may be attributed to the partial oxidation of metallic silver particles, which is inevitable for the resultant samples in contact with air.

**Figure 4:**
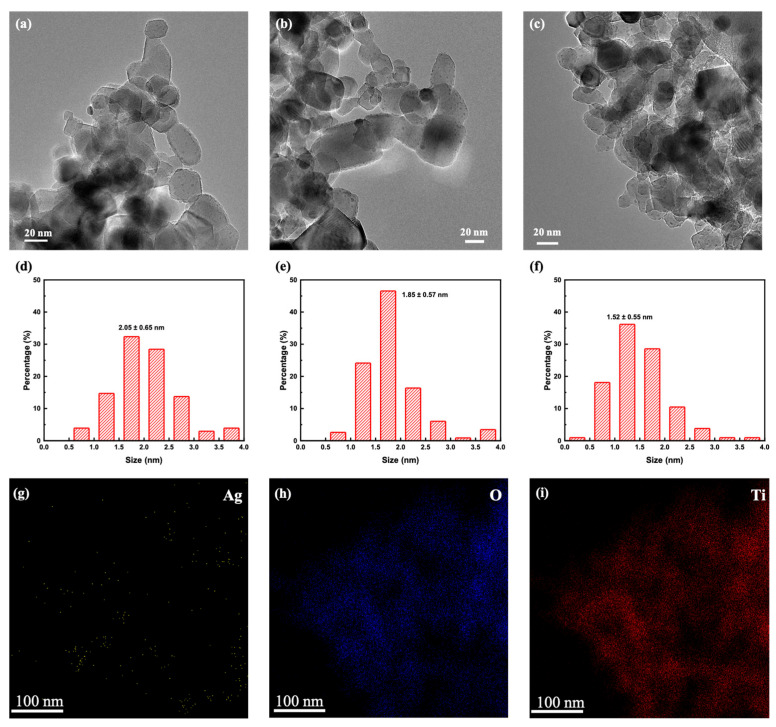
TEM images of Ag/P25 nanocomposites prepared at different dose rates and their corresponding particle size histograms: Ag/P25-5 (**a**,**d**); Ag/P25-3 (**b**,**e**); Ag/P25-6 (**c**,**f**); (**g**–**i**) the elemental mappings of Ag/P25-6 of Ag, O, Ti, respectively.

**Figure 5:**
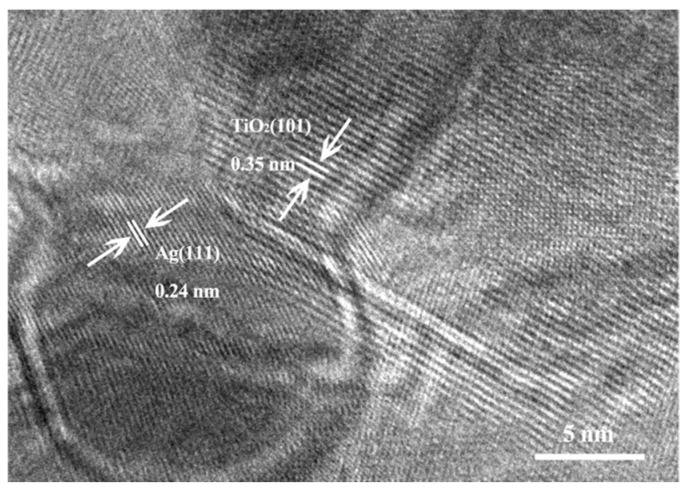
HRTEM image of Ag/P25-3.

TEM measurements were applied to detect the distribution and particle size of Ag NPs of Ag/P25 nanocomposites. TEM images in Figure 4a–c reveal that Ag NPs were loaded on the surface of P25 after gamma-ray irradiation, and that these Ag NPs at different dose rates are similar in shapes and locations but differ in particle size distributions. Figure 4d–f show the average diameters of Ag NPs of Ag/P25-5, Ag/P25-3, and Ag/P25-6. As the dose rate increases from 7.2 Gy/min, 31 Gy/min to 130 Gy/min, the average particle sizes decrease from 2.05 nm, 1.85 nm to 1.52 nm, respectively, and it is highly intuitive to notice that fewer outstanding large clusters of Ag NPs are observed under a higher dose rate. The morphology of Ag/P25 nanocomposites was examined by HRTEM, as shown in Figure 5. The lattice spacings of 0.35 nm and 0.24 nm match the crystal lattices of TiO_2_ and Ag and can be attributed to anatase TiO_2_ (101) and cubic Ag (111) species, respectively. Furthermore, the elemental mapping pictures of Ag/P25-6 (shown in Figure 4g–i) confirm the presence of the small dopped dots as Ag NPs.

**Figure 6:**
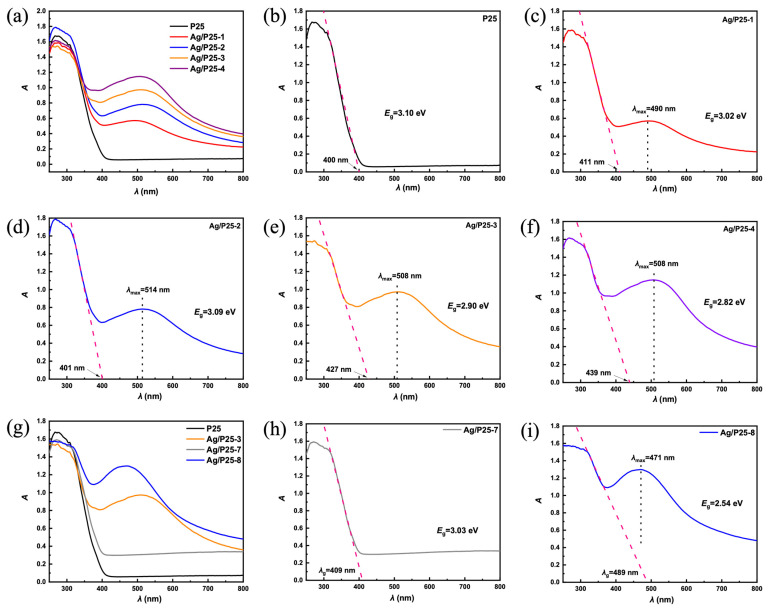
UV-Vis DR spectra of: (**a**,**g**): P25 and as-prepared Ag/P25 nanocomposites; (**b**): P25; (**c**–**f**): Ag/P25 nanocomposites synthesized at different feed ratios, Ag/P25-1, Ag/P25-2, Ag/P25-3, and Ag/P25-4, respectively; (**h**,**i**): Ag/P25 nanocomposites synthesized with different free radical scavengers, EMImAc, EG.

The band gap width between the valence band and conduction band of a semiconductor material is a crucial parameter because it determines the potential energy difference between the electrons and the holes, which can be measured from the UV-Vis diffuse reflectance (DR) spectra. Figure 6 shows the UV-Vis DR spectra of P25 and different catalysts with various amounts of Ag and free radical scavengers. The cutting-line method is a common band gap calculation method whose principle is that the band edge wavelength λ_g_ is determined by the band gap width E_g_, E_g_ = 1240/λ_g_ (nm) [71]. The *x* value of the tangent of the absorbance curve intersected with the *x*-axis is recorded as the band edge wavelength λ_g_. UV-Vis DR spectrum of P25, shown in Figure 6b, exhibits an absorption band at λ_g_ = 400 nm, indicating its band gap width of 3.10 eV, which is consistent with previous research [72]. For Ag/P25 photocatalysts prepared at different Ag/P25 feed ratios shown in Figure 6c–f, there are other wide absorption bands centered at 490–515 nm. The appearance of these absorption bands in the visible light range can be attributed to the LSPR effect of Ag NPs doped on the surface of P25. As the feed ratio of Ag/P25 increases, both the peak intensity and half width increase, indicating that higher concentrations of silver nitrate led to the formation of more clusters of Ag NPs. On the other hand, the band gap widths of catalysts decrease with increasing Ag content because of the enhancement of the LSPR effect. For catalysts synthesized with different free radical scavengers, their band gap widths are smaller than P25, and band edge wavelengths extend to the visible light range, which can be attributed to the doped Ag NPs [27,42].

### 3.2. Photocatalytic Activities

**Figure 7:**
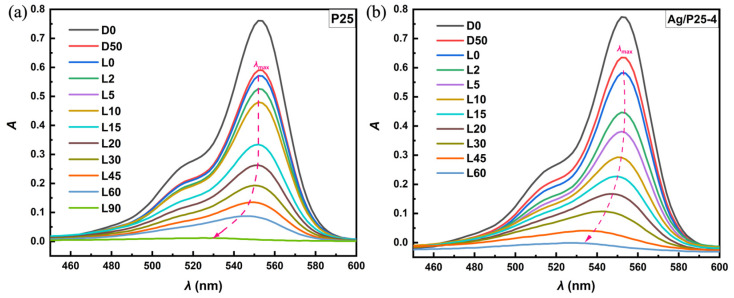
Absorption spectral changes of RhB solutions in the presence of (**a**) P25 and (**b**) Ag/P25-4 at given times. DX: time t = X(min) since dark reaction started; LX: time t = X(min) since Xenon light irradiation.

The photocatalytic performances of the as-prepared Ag/P25 nanocomposites were measured under Xenon light (λ > 200 nm). Figure 7 illustrates the changes in UV-Vis absorption spectra of RhB solutions in the presence of P25 and Ag/P25-4 at predetermined times. The dotted arrow indicates the blue shift in the maximum absorption wavelength (λ_max_) of RhB solutions during the photodegradation, which implies the de-ethylation process of RhB [39]. Previous studies by Natarajan et al. [73] and Liang et al. [61] have reported that λ_max_ of the oxidation products of RhB under UV and visible light irradiation are gradually decreasing though the de-ethylation process. The RhB solutions with catalysts can reach the absorption–desorption equilibrium in the dark for 60 min, and almost complete degradation of RhB molecules was achieved after a 60-min Xenon light irradiation.

**Figure 8:**
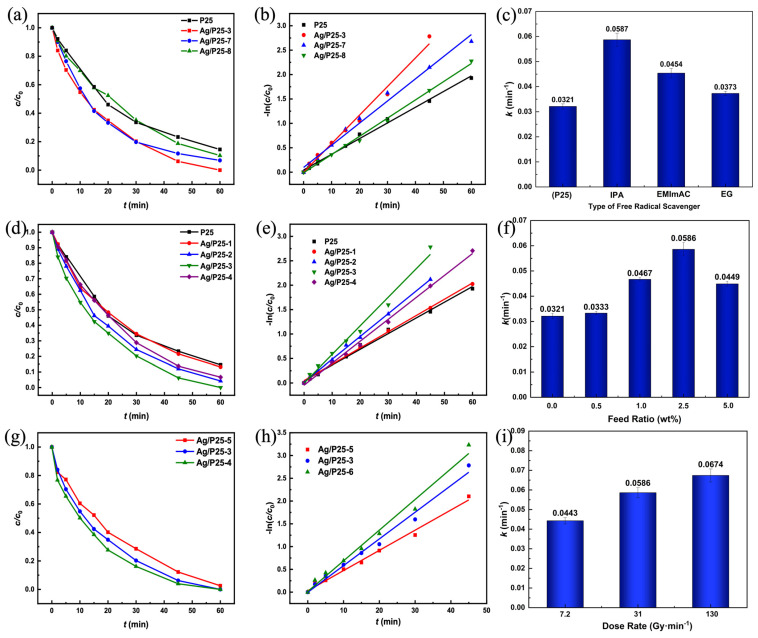
(**a**,**d**,**g**): Comparisons of the photocatalytic degradation curves of RhB (*c*/*c*_0_ vs. irradiation time) of different Ag/P25 nanocomposites; (**b**,**e**,**h**): plots of −ln(*c/c*_0_) versus irradiation time; (**c**,**f**,**i**): comparisons of reaction rate constants under different synthesis conditions.

The photocatalytic degradation curves of RhB (*c*/*c*_0_ vs. irradiation time) are presented in Figure 8a,d,g, revealing that the degradation rates of RhB with Ag/P25 nanocomposites (calculated based on Equation (1)) are higher than that with P25. A pseudo-first order kinetics fitting (Equation (2)) is performed to draw the plots of −ln(*c*/*c*_0_) vs. irradiation time and determine the reaction rate constant k. The corresponding plots of photocatalysts synthesized with different free radical scavengers, Ag/P25 feed ratios, and dose rates are depicted in Figure 8b,e,h and Figure 8c,f,i, respectively. The squared correlation coefficients of linear fitting are all greater than 0.985, indicating that there is a strong linear relationship between −ln(*c*/*c*_0_) and irradiation time, and that the variation of −ln(*c*/*c*_0_) versus time is in accordance with the first order reaction. Applying IPA, EMImAc, and EG as different free radical scavengers in Ag/P25-3, Ag/P25-7, and Ag/P25-8, the order of their rate constants is Ag/P25-3>Ag/P25-7>Ag/P25-8. Therefore, isopropyl alcohol is selected to act as the free radical scavenger in further studies of this work. With Ag/P25 feed ratios increasing from 0.5%, 1.0%, 2.5% to 5.0%, the rate constants first increase and then decrease, reaching the peak at 2.5 wt% Ag/P25 feed ratio, and they are better than that of P25. This result indicates that Ag NPs significantly influence the photocatalytic activities of Ag/P25 nanocomposites. Regarding the influence of different dose rates, the rate constants of Ag/p25-5, Ag/P25-3, and Ag/P25-6 become greater while the dose rates increase from 7.2 Gy/min to 130 Gy/min. Among the eight synthesized Ag/P25 nanocomposites, Ag/P25-6 exhibits the best photocatalytic performance under Xenon light irradiation, with a rate constant of 0.0674 min^−1^, which is 2.1 times higher than that of P25.

Other investigations have also explored the photocatalytic performance of Ag/TiO_2_ nanocompounds in degrading RhB (shown in Table 3). For instance, Sun et al. [53] synthesized Ag-TiO_2_ nanowire at a mole ratio of 3% (3 at%) and utilized 100 mg samples to photodegrade 200 mL RhB solution (10 mg/L), achieving complete removal of RhB in 45 min. Hajipour et al. [74] developed another variant of Ag/TiO_2_ nanowire and utilized it to break down a 12 mg/L RhB solution, with a rate constant of 0.026 min^−1^. In contrast, Ag/P25-6, in our study, displayed a much higher rate constant of 0.0674 min^−1^ and required less photocatalyst (30 mg) to degrade a higher concentration of RhB solution (30 mg/L), showcasing superior photocatalytic performance when compared to previous works.

**Figure 9:**
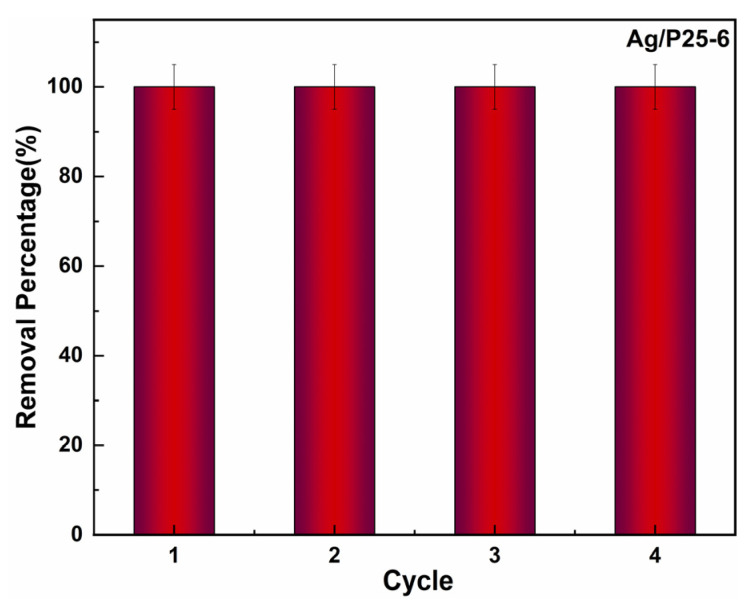
Recycling photocatalytic performance of Ag/P25-6 under Xenon light irradiation.

The recycling stability is also an important parameter for assessing the catalysts, as high photocatalytic performance may be confronted with various risks such as the shedding of Ag NPs during degradation process, which can cause a sudden decrease in photocatalytic activities. To investigate the recycling stability, it is necessary to conduct recycling experiments. Figure 9 displays the removal percentages of RhB under different catalytic cycles. After four cycles, RhB can be completely removed with Ag/P25-6 as the catalyst under 60 min Xenon light irradiation. The result clearly indicates that Ag/P25-6 exhibited a stable recycling photodegradation performance with a high rate, suggesting its outstanding photocatalytic recycling stability.

### 3.3. Mechanism Clarification

**Figure 10:**
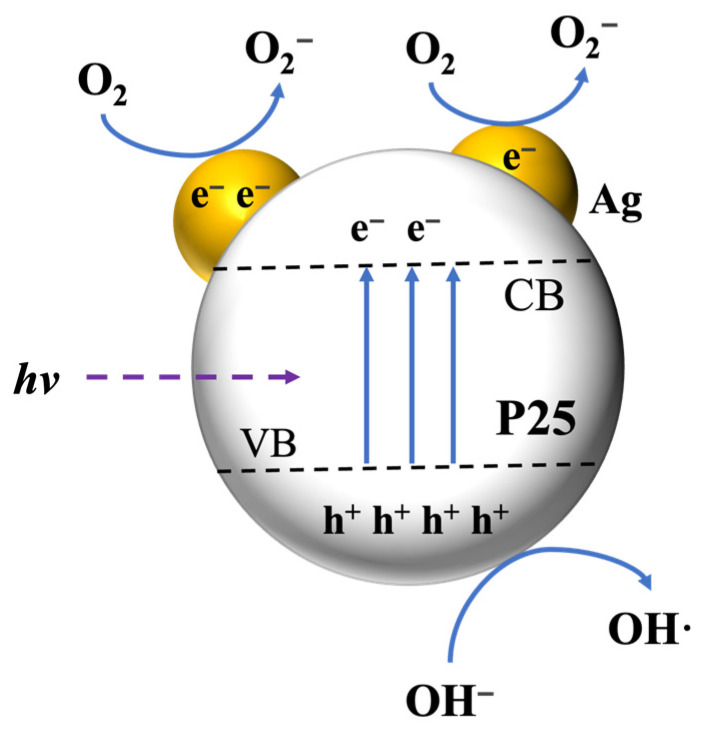
Schematic diagram of Ag/P25 nanocomposites for degrading RhB under Xenon light.

The improved photocatalytic performance of Ag/P25 nanocomposites over P25 can be attributed to the enhancement of electron–hole separation and light utilization efficiency. Under Xenon light exposure (Figure 10), the valence electrons of P25 become excited and transfer to the conduction band, and Ag NPs serve as electron traps to facilitate the electrons to participate in the reductive reaction of O_2_ to O_2_**^−^**, as well as to prevent the recombination of electrons and holes [39,40]. Meanwhile, the LSPR effect of Ag NPs under visible light generates electron–hole pairs on their surface [61]. The photogenerated electrons from the LSPR effect then transfer to the conduction band of P25, creating a rich electronic state as the energy level of P25 is lower than that of Ag NPs [42]. As a result, compared to pure P25, Ag/P25 nanocomposites make better use of Xenon light, enabling photogenerated electrons to transfer and participate in the reaction, thereby significantly enhancing the photocatalytic activity.

In this work, Ag/P25-6, which was synthesized with a feed ratio of 2.5 wt%, an irradiation dose rate of 130 Gy/min, and IPA as the free radical scavenger, exhibited the best photocatalytic activity among all Ag/P25 nanocomposites. The impact of feed ratio on the photocatalytic performance can be explained from the perspective of electron–hole separation and recombination. A feed ratio below 2.5 wt% does not provide sufficient Ag NPs on the surface of P25 to serve as electron traps, limiting the efficiency of electron–hole separation and reducing the reaction rate of RhB. Conversely, a high feed ratio increases the distribution density of Ag NPs on the surface of P25, causing overlapping surface plasmon resonance regions of Ag NPs, proceeding the electron–hole recombination process and reducing the reaction efficiency [42,53,75].

The influence of dose rate on the photocatalytic activity can be understood by the size of Ag NPs. From previous research by Flores-Rojas et al. [63], given a certain dose irradiation, lower dose rate induces larger particle size of Ag NPs because the production rate of reducing free radicals is slower than the association of ions with atoms. As shown in TEM image (Figure 3a–c), Ag NPs are formed in the shape of sphere, and the reduced particle sizes lead to bigger specific surface area and contact area with RhB and greatly enhance the surface-to-volume ratio of photocatalysts, which hence would improve the photocatalytic activity of Ag/P25 nanocomposites [40,47].

## 4. Conclusions

In summary, we have reported the successful synthesis of an efficient photocatalyst of Ag/P25 nanocomposites by utilizing a one-step gamma-ray induced reduction method in aqueous solution. The gamma-ray irradiation allowed successful doping of Ag NPs onto the surface of P25, where the dose rate had a significant impact on the size and distribution of Ag NPs, as well as the photocatalytic activity of Ag/P25 nanocomposites. Additionally, the feed ratio of Ag/P25 and the choice of free radical scavenger were found to influence the photocatalytic activity of the resulting nanocomposites. Notably, Ag/P25-6, synthesized with a feed ratio of 2.5 wt%, a dose rate of 130 Gy/min, and IPA as the free radical scavenger, exhibited a reaction rate constant 2.1 times higher than that of P25 due to its suitable load amount and small particle size. Furthermore, under Xenon light irradiation, Ag/P25 nanocomposites demonstrated superior photocatalytic activity compared to P25 due to the enhanced electron–hole separation and the extended responding range to the visible light spectrum caused by the LSPR effect. This study offers an effective method for the synthesis of Ag/TiO_2_ nanocomposites with efficient photocatalytic performance, which could be potentially extended to the synthesis of other metal-doped semiconductor catalytic system.

## Figures and Tables

**Table 1:** Sample abbreviations and corresponding synthesis details.

Sample	Feed Ratio (Ag/P25 wt%)	Dose Rate (Gy/min)	Free Radical Scavenger
P25	0	/	/
Ag/P25-1	0.5	31	IPA
Ag/P25-2	1.0	31	IPA
Ag/P25-3	2.5	31	IPA
Ag/P25-4	5.0	31	IPA
Ag/P25-5	2.5	7.2	IPA
Ag/P25-6	2.5	130	IPA
Ag/P25-7	2.5	31	EMImAc
Ag/P25-8	2.5	31	EG

**Table 2:** ICP-OES results of samples with different Ag/P25 feed ratios.

Sample	Feed Ratio (Ag/P25 wt%)	Ag (wt%)	Doping Efficiency (%)
Ag/P25-1	0.5	0.37	74
Ag/P25-2	1.0	0.71	71
Ag/P25-3	2.5	2.18	87
Ag/P25-4	5.0	4.22	84

**Table 3:** Comparison with other Ag/TiO_2_ photocatalysts.

Photocatalysts	Preparation Method	Light Source	Reaction Solution and Amount	Removal Time and Percentage	Reaction Rate Constant (min^−1^)	Reference
Ag-1%@P25	Photo reduction	Homemade light source (λ > 400 nm, ~150 mW/cm^2^)	10 mg/L, 25 mL, 50 mg	30 min, 95%	0.113(4)	[42]
3 at% Ag-TiO_2_ nanowire	Hydrothermal process	350 W Xenon light	10 mg/L, 200 mL, 100 mg	45 min, 100%	NA	[53]
2 at% Ag-TiO_2_ nanostructure	Hydrothermal process	800 W Xenon light (λ > 420 nm)	10 mg/L, 50 mL, 30 mg	270 min, 95%	0.01108	[54]
Ag/TiO_2_-II	Photo reduction	500 W mercury lamp (UV)	10 mg/L, 300 mL, 600 mg	180 min, 93%	0.0144	[61]
		500 W Xenon lamp (Visible light)		180 min, 88%	0.0111	
Ag/TiO_2_ nanowire	Polyol method	Xenon light of 75.9 kJ/m^2^	12 mg/L, NA	100 min, 84%	0.026	[74]
1.0%Ag–TiO_2_/ SBA-16	Wet impregnation method	300 W Xenon light (λ > 420 nm)	10 mg/L, 100 mL, 50 mg	120 min, 90%	0.02072	[75]
Ag/P25-6	Gamma radiation reduction	300 W Xenon light	30 mg/L, 90 mL, 30 mg	45 min, 97%	0.0674	this work

## Data Availability

Not applicable.
